# Tilapia-waste flour as a natural nutritional replacer for bread: A consumer perspective

**DOI:** 10.1371/journal.pone.0196665

**Published:** 2018-05-03

**Authors:** Maria Lúcia G. Monteiro, Eliane T. Mársico, Manoel S. Soares Junior, Rosires Deliza, Denize C. R. de Oliveira, Carlos A. Conte-Junior

**Affiliations:** 1 Department of Food Technology, University Federal Fluminense, Niterói, Rio de Janeiro, Brazil; 2 Institute of Chemistry, University Federal of Rio de Janeiro, Rio de Janeiro, Brazil; 3 Department of Food Engineering, School of Agronomy, University Federal of Goiás, Goiânia, Brazil; 4 Embrapa Food Technology, Rio de Janeiro, Brazil; University of Illinois, UNITED STATES

## Abstract

Six bread formulations with different levels of tilapia-waste flour (BTF0%, BTF2.5%, BTF5%, BTF10%, BTF15%, and BTF20%) were analyzed for nutritional composition and sensory characterization. Tilapia-waste flour (TF) increased (*P* < 0.05) the lipid, protein and ash contents, and decreased (*P* < 0.05) the levels of carbohydrates and total dietary fiber. BTF0%, BTF2.5% and BTF5% received the highest (*P* < 0.05) scores for acceptance and preference. Despite this apparent consumer preference for low or no levels, TF can be added to bread at levels below 12.17% (*P* < 0.05) without triggering consumer rejection. TF changed (*P* < 0.05) the sensory characterization of bread because of a disagreeable flavor, aroma, and texture; however, airy appearance, sticky in the teeth and cream color did not influence the overall liking. TF at 5% enhanced the nutritional value while maintaining acceptable sensory scores for bread, constituting a potential strategy to satisfy consumer and industry requirements.

## Introduction

Consumer demand for healthier foods has become increasingly widespread in recent years [1]. Bread manufactured with refined wheat flour is a low-cost staple food that is well accepted worldwide [2]. However, it is rich in carbohydrates, mainly highly digestible starch, with a high glycemic index (GI) [3]. A high GI may be associated with increased risk of diabetes [4] and biliary-tract cancer [5]. Moreover, bread is also low in protein [2]. However, as bread is a good carrier of functional ingredients, a wide variety of studies have reported nutritional improvement after replacement of wheat flour by different industry by-products as fish flour or powder from several seafood sources, including tilapia [6], saithe surimi [7], red-tailed Brycon (*Brycon cephalus*) [8], and shrimp [9]. However, the addition of by-product ingredients in traditional bakery products may cause sensory changes and consumer rejection [10]. To the best of our knowledge, no study has evaluated the consumer perception of bread enriched with Nile tilapia-waste flour.

Although Nile tilapia (*Oreochromis niloticus*) is one of the most commercially important freshwater fish species, contributing approximately 9% of the total amount of fish produced globally [11], it has a low fillet yield of only about 30% of the live weight, and therefore most of processed tilapia is considered waste [12]. According to Castro-Muñoz et al. [13], the high cost of waste disposal has impelled the food industry to develop waste-recycling techniques to decrease environmental pollution and improve profits. Among fish byproducts, tilapia flour manufactured from meat adhered to skin and bones yields about 8% of the whole-fish weight, and is considered an inexpensive source of essential nutrients, constituting an interesting alternative nutritional supplement for bakery products [12,14].

Considering the market requirements, one of the main challenges of the food industry is to produce convenience food with added nutritional value, accessible cost, and pleasant sensory properties. Also, there is a lack of understanding of consumer perceptions about bread enriched with fish-waste flour, which is important to encourage the healthy-food market based on fish waste from processing. This study (1) characterized formulations of bread fortified with different levels of tilapia flour, from the point of view of nutritional value and sensory attributes; (2) determined the overall liking for all bread formulations; and (3) determined the cutoff point (COP) for wheat flour replacement by tilapia-flour in wheat bread, using a consumer-based approach.

## Materials and methods

### Tilapia flour preparation

A total of 9.0 ± 0.3 kg of mechanically separated meat (MSM) of tilapia, packed in polyethylene bags, was purchased from a commercial fish farm (Cachoeiras de Macacu, Rio de Janeiro, Brazil). The MSM was dried for 12 h at 65°C in a forced-air convection oven (TE-394/3, Tecnal, Piracicaba, São Paulo, Brazil) in the bakery pilot plant, to obtain the tilapia flour.

### Bread production

Bread formulations were prepared according to the protocol described by Stokić et al. [15] with slight modifications. Each of six formulations, consisting of refined white wheat flour and tilapia-waste flour (TF) in different proportions, was added to 40 g baker's yeast (Fleischmann’s^®^, ACH Food Companies, Inc., Pederneiras, São Paulo, Brazil), 50 g sugar (União^®^, São Paulo, Brazil), 20 g salt (Cisne^®^, São Paulo, Brazil), 50 g dough improver (Fleischmann’s^®^, ACH Food Companies, Inc., Pederneiras, SP, Brazil), 33 g vegetable fat (Primor^®^, São Paulo, Brazil), and 640 mL water (Table 1). The wheat flour replacement by TF was 0%, 5%, 10%, 20%, 30%, and 40%, representing final bread formulations with 0%, 2.5%, 5%, 10%, 15%, or 20% TF, which were termed BTF0%, BTF2.5%, BTF5%, BTF10%, BTF15%, and BTF20%, respectively. All ingredients were purchased in a bakery-products store (Torres Alimentos Ltda., Santa Genoveva, Goiás, Brazil). The TF was mixed with 240 mL of water, and after 15 min this content was added to the mixed ingredients (refined white wheat flour, baker's yeast, sugar, salt, dough improver and vegetable fat). Four hundred mL of water was slowly added (in 100 mL increments to complete 400 mL) to form a homogeneous dough, which was placed in a dough mixer (BP-5, Gastromaq, Rio Grande do Sul, Brazil) set at position five for 20 min. After mixing, each dough formulation was divided into three equal portions, manually kneaded for 20 min at 25°C, sheeted, and rolled. The dough was covered with cloth, allowed to rest at 25°C for 30 min, baked at 180°C for approximately 25 min in a baking oven (FERI-90, Venancio Aires, Rio Grande do Sul, Brazil), and cooled at room temperature to 25°C. The breads were packed in high density polyethylene bags, and immediately analyzed for nutritional and sensory characterization.

**Table 1:** Bread formulations with different tilapia flour levels.

Ingredients	Formulations					
	BTF0%	BTF2.5%	BTF5%	BTF10%	BTF15%	BTF20%
Wheat flour (g)	840	798	756	672	588	504
Tilapia flour (g)	0	42	84	168	252	336
Baker's yeast^*^ (g)	40	40	40	40	40	40
Sugar (g)	50	50	50	50	50	50
Salt (g)	20	20	20	20	20	20
Dough improver^¥^ (g)	50	50	50	50	50	50
Vegetable fat (g)	33	33	33	33	33	33
Water (mL)	640	640	640	640	640	640

BTF0%, BTF2.5%, BTF5%, BTF10%, BTF15%, and BTF20% means bread with tilapia flour at 0%, 2.5%, 5%, 10%, 15%, and 20% (w/w), respectively.

^*^Baker's yeast composition: *Saccharomyces cerevisiæ* and sorbitan monostearate.

^¥^Dough-improver composition: maize starch (*Bacillus thuringiensis*, *Streptomyces viridochromogenes*, *Agrobacterium tumefaciens*), sugar, polysorbate 80, ascorbic acid, azodicarbonamide, and alpha-amylase.

### Proximate composition, energy content, and total dietary fiber

The contents of moisture (AOAC method 950.46B), lipid (AOAC method 991.36), protein (AOAC method 955.04; N × 6.25 and 5.70 for bread manufactured with and without tilapia flour, respectively), ash (AOAC method 920.153), and total dietary fiber (AOAC 991.43) were determined for the six bread formulations described in Table 1, following the procedures of the Association of Official Analytical Chemists [16]. The carbohydrate content was evaluated by calculating the percent remaining to 100% after all the other components (moisture, protein, ash, and lipid) were measured. The energy value was calculated by the formula: energy value (kcal/100g) = 4 × protein (%) + 9 × lipid (%) + 4 × carbohydrate (%) [17]. These analyses were carried out in duplicate (n = 2) for each bread formulation totaling 18 sample units.

### Consumer study

#### Participants

One hundred consumers (untrained panelists; n = 100) were invited to participate in the study. They were recruited among workers and students at Embrapa Food Technology (Rio de Janeiro, Brazil), according to their interest in and availability to participate in the study. All participants consumed bread regularly. Their socio-demographic characteristics are shown in Table 2. Participants signed an informed consent form and received a small gift for their participation. The study was approved by the Research Ethics Committee of the Universidade Federal Flumimense (protocol number 33733914.4.0000.5243, Niterói, Rio de Janeiro, Brazil).

**Table 2:** Demographic characteristics of the participants (n = 100).

Characteristics	%
*Gender*	
Female	64
Male	36
*Age (years)*	
18–25	10
26–35	31
36–45	29
46–55	17
56–65	11
66 and older	1
*Education*	
Incomplete high school	1
Complete high school	3
Incomplete undergraduate	9
Complete undergraduate	11
Complete graduate	75
*Household income*^¥^	
1–5	18
> 5–10	26
> 10–20	36
> 20–30	13
> 30	6
*Bread consumption frequency*	
Never	0
Rarely	4
Frequently	14
Daily	67
More than once a day	14

^¥^The household income was based on Brazilian monthly minimum wage (BMW; $ 259 in November 2016).

### Sample preparation

Each bread formulation described in Table 1 was sliced (1 cm thick) and cut into four pieces. A piece of each formulation was presented individually to the participants, in a white paper napkin labeled randomly with a 3-digit code, following the balanced presentation order.

### Experimental procedure

The study comprised affective tests (acceptance, intention to consume, and intention to purchase) and a descriptive evaluation using check-all-that-apply (CATA) questions. The socio-demographic characteristics of consumers were also noted.

Participants evaluated the acceptability of the bread formulations using a 9-point structured hedonic scale, ranging from 1: dislike extremely to 9: like extremely. Next, they were asked to try the sample and to answer “yes” or “no” to the following questions, to establish the sensory COP of wheat flour replacement by tilapia-flour in wheat bread by the survival-analysis method [18]:

a) “Suppose that you bought this product to eat or that it was served to you in your home. Would you consume it?”
b) “Suppose that this product is new on the market. Would you buy it?”

After that, the sensory characteristics were evaluated using CATA questions [19]. The CATA terms were previously defined with six experienced assessors in the field of sensory perception and generation of attributes. Bread formulations with 7.5% and 17.5% tilapia flour were used as stimuli in this stage, and 24 terms were identified. The CATA terms were compact appearance, airy appearance, cream color, light color, dark color, yeast aroma, bread aroma, strong aroma, odd aroma, cheese aroma, odd flavor, cheese flavor, yeast flavor, salty taste, acid taste, hard, soft, compact texture, moist, crumbly, raw, sticky in the teeth, oily, and spongy. The presentation order of the CATA terms was balanced in the questionnaire for each sample and each participant [20].

Finally, the participants answered the last question “Would you be interested in eating bread with a higher amount of proteins and minerals?” Prior to the analysis, no information about the aim of the study was provided to participants. Water at room temperature and unsalted crackers were served to participants to refresh their sense of taste between samples.

### Statistical analysis

Analysis of variance (ANOVA) and Tukey’s test (*P* < 0.05) were performed on the data for proximate composition, energy value, total dietary fiber, and hedonic scores, to determine differences among all the bread formulations. In addition, Internal Preference Mapping was carried out to detect consumer preferences among the different formulations. For the questions, the answers were categorized as 0 (no) and 1 (yes), and survival-analysis statistics (Weibull model for Question a, and log-normal model for Question b) with a 50% rejection probability [21] were applied in order to determine the sensory COP of added tilapia flour in bread. The Correspondence Analysis (CA) was performed on the frequency of mentions of each CATA term for each sample, and the differences (*P* < 0.05) among bread formulations related to each CATA term included in the questionnaire were identified by Cochran’s Q test. All data were submitted to Multifactorial Analysis (MFA) to determine the parameters that were influenced by replacement of wheat flour with tilapia-flour. The demographic data and the final question were evaluated by the frequency of each response. All statistical analyses were carried out using R language [22] with a 95% confidence interval.

## Results and discussion

### Proximate composition, energy content, and total dietary fiber

The substitution of wheat flour by tilapia flour increased (*P* < 0.05) the lipid, protein, and ash contents, and decreased (*P* < 0.05) the levels of carbohydrate and total dietary fiber (Table 3). BTF15% and BTF20% had the highest (*P* < 0.05) moisture content and the lowest (*P* < 0.05) energy value. No differences (*P* > 0.05) were observed in moisture contents and energy values among other tilapia-bread formulations at 0%, 2.5%, 5%, and 10%. These results are strongly related to the wheat and tilapia flour compositions. Tilapia flour has a low carbohydrate level (< 1.5%) and high levels of protein (> 45%), lipid (> 25%), and ash (> 3%) [12], while the wheat flour counterpart contains higher levels of carbohydrate (> 75%) and fiber (> 10%), and lower levels of protein (< 11%), lipid (≤ 1.5%), and ash (≤ 0.38%) [23,24]. Although wheat flour (13.50%) has a higher moisture content than tilapia flour (7.84%) [12,24], the results of this study can be attributed to differences in protein conformation, amino acid composition, and surface polarity/hydrophobicity between wheat and fish, leading to different water-binding capacities [23,25–27]. Our findings for energy value are attributable to changes in the lipid, protein, and carbohydrate contents due to use of tilapia-flour in substitution to wheat flour, together with their respective individual weights in the formula proposed by Merrill and Watt [17]. Similar nutritional compositions of bread enriched with different protein sources were found by Adeleke and Odedeji [6], Bastos et al. [8], and Jeyakumari et al. [9].

**Table 3:** Proximate composition (%), energy value (kcal/100 g) and total dietary fibers (%) of bread formulations with different tilapia flour levels.

Parameters	Formulations					
	BTF0%	BTF2.5%	BTF5%	BTF10%	BTF15%	BTF20%
Moisture	36.09±1.43^b^	34.82±1.12^b^	36.44±0.48^b^	37.16±0.21^b^	42.74±0.76^a^	42.39±0.81^a^
Protein	6.78±0.60^e^	8.71±0.54^d^	10.83±0.34^c^	12.01±0.35^c^	14.36±0.40^b^	16.28±0.28^a^
Lipid	1.39±0.07^e^	1.85±0.00^d^	2.51±0.12^c^	2.67±0.10^c^	2.97±0.13^b^	3.52±0.10^a^
Ash	1.93±0.01^d^	1.82±0.01^d^	2.13±0.08^c^	2.29±0.04^b^^c^	2.34±0.02^b^	2.89±0.06^a^
Carbohydrate	53.93±1.95^a^	52.70±1.65^a^^b^	48.10±0.10^b^^c^	45.88±0.08^c^	37.61±1.31^d^	34.93±0.57^d^
Energy value	255.33±6.04^a^	262.27±4.44^a^	258.27±2.84^a^	255.57±0.16^a^	234.55±2.44^b^	236.50±1.07^b^
Total dietary fibers	6.84±0.28^a^	5.82±0.14^b^	5.86±0.14^b^	5.79±0.11^b^	5.85±0.23^b^	5.87±0.16^b^

BTF0%, BTF2.5%, BTF5%, BTF10%, BTF15%, and BTF20% means bread with tilapia flour at 0%, 2.5%, 5%, 10%, 15%, and 20% (w/w), respectively. ^a─e^Different superscripts indicate differences (*P* < 0.05) among formulations. Results are expressed as means ± standard deviation (n = 2).

### Acceptance evaluation

BTF0%, BTF2.5%, and BTF5% received the highest (*P* < 0.05) liking scores among samples, BTF10% and BTF15% received intermediate acceptance (*P* < 0.05), while BTF20% received the lowest (*P* < 0.05) overall liking score. No difference (*P* > 0.05) was observed among BTF0%, BTF2.5%, and BTF5%, and between BTF10% and BTF15% for hedonic scores related to overall liking. Similarly to our study, other authors also reported successful acceptance after addition of 5% fish-protein concentrate in biscuits [28], shrimp protein hydrolysate at 5–7.5% in extruded products [9], and fish powder at 3–7% in snacks [7].

### Internal preference mapping

**Fig 1:**
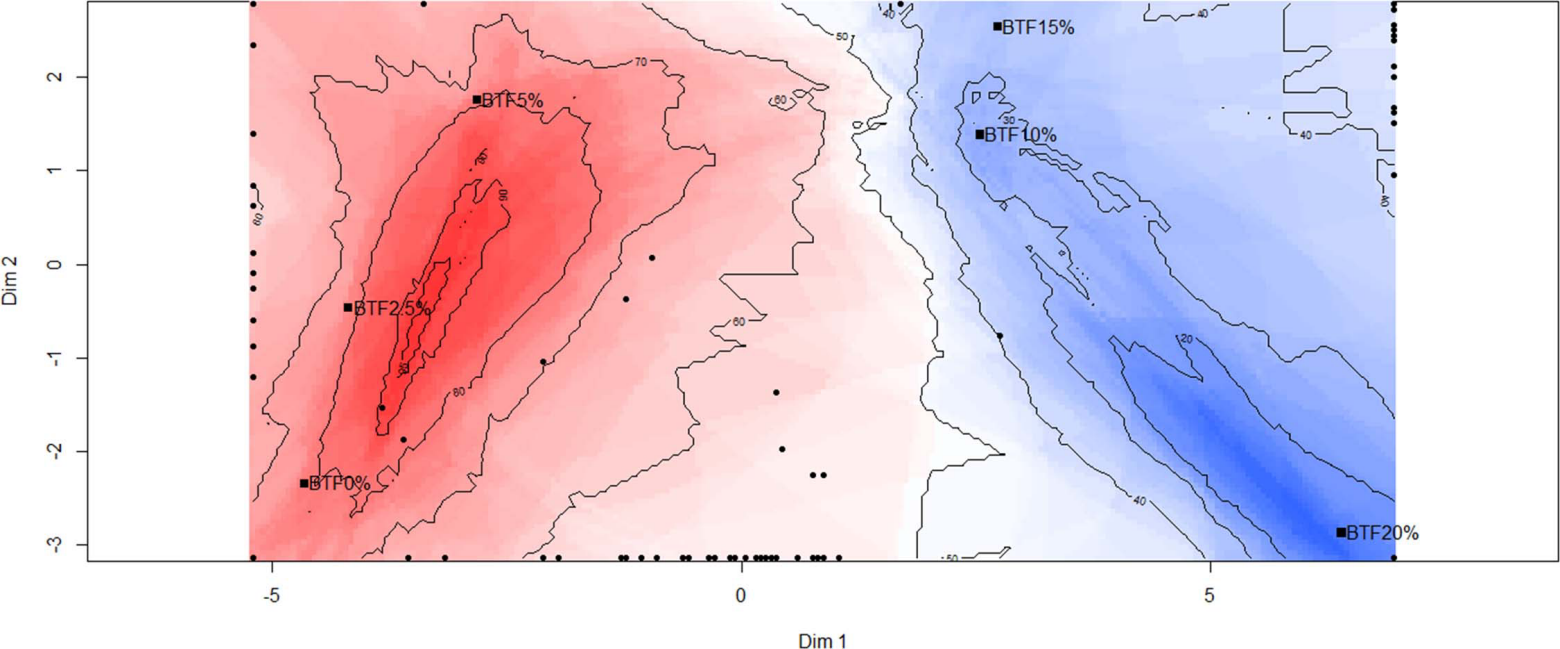
Color counter plot of the average overall liking scores by consumers (n = 100) evaluating BTF0%, BTF2.5%, BTF5%, BTF10%, BTF15%, and BTF20%. BTF: bread with tilapia-waste flour at 0%, 2.5%, 5%, 10%, 15%, and 20% (w/w), respectively. Red areas indicate samples with higher overall liking.

The internal preference mapping analysis revealed that the majority of the participants preferred the bread with 0% and 2.5% TF, followed by the formulation with 5% TF. Bread formulations with higher amounts of TF (10%, 15%, and 20%) were less preferred by participants, especially BTF20% (Fig 1). The inclusion of high levels of fish sources in foodstuffs is problematic, due to the fishy flavor and odor generated mainly by free fatty acids and volatile sulfur compounds [29]. Despite this, protein enrichment of commercial products may be advantageous, depending mainly on product processing, seafood type, and the proportion used [30]. Similar consumer responses were reported for bread enriched with up to 8.4% red-tailed Brycon flour [8], a corn snack supplemented with 7% *Pollachius virens* protein powder [7], and a snack enriched with shrimp protein hydrolysate and shrimp powder at 5–7.5% [9]. On the other hand, Fitzgerald et al. [31] reported that bread enriched with 4% *Palmaria palmata* protein hydrolysate was less preferred, due to the strong bitter taste.

### Check-all-that-apply (CATA) questions

The tilapia flour in substitution to wheat flour changed 12 sensory attributes: *cream color*, *moist*, *crumbly*, *salty taste*, *hard*, *raw*, *acid taste*, *sticky in the teeth*, *cheese flavor*, *oily*, and *cheese aroma* (Table 4). The airy appearance was increased by small amounts of added tilapia flour (up to 5%), and decreased in formulations enriched with larger amounts (10%, 15%, and 20%).

**Table 4:** Average overall liking scores and frequency of the CATA terms used by consumers (n = 100) for all bread formulations with different tilapia flour levels.

Terms	BTF						Terms	BTF					
	0%	2.5%	5%	10%	15%	20%		0%	2.5%	5%	10%	15%	20%
Overall liking^¥^	6.3^a^	6.3^a^	6.0^a^	4.6^b^	4.4^b^	3.3^c^							
Cream color***	**18**	**29**	**35**	**37**	**43**	**21**	Cheese aroma***	**5**	**7**	**18**	**28**	**21**	**34**
Clear color	74	64	52	22	18	9	Yeast flavor	23	19	20	31	28	27
Dark color	0	1	1	25	26	60	Odd flavor	7	7	19	46	50	58
Compact appearance	38	25	19	57	48	69	Cheese flavor***	**6**	**9**	**32**	**40**	**33**	**41**
Airy appearance***	26	28	40	12	17	4	Salty taste***	**10**	**15**	**17**	**27**	**26**	**28**
Moist*	**14**	**20**	**22**	**21**	**33**	**26**	Acid taste**	**2**	**2**	**6**	**11**	**7**	**15**
Crumbly***	**10**	**22**	**34**	**42**	**39**	**40**	Compact texture	36	25	25	49	60	72
Raw***	**5**	**7**	**12**	**16**	**22**	**27**	Hard***	**8**	**7**	**0**	**5**	**5**	**28**
Yeast aroma	24	28	25	30	32	33	Soft	60	68	58	34	38	14
Bread aroma	64	60	44	13	10	2	Sticky in the teeth*	**22**	**23**	**34**	**29**	**29**	**18**
Strong aroma	6	6	7	25	28	48	Spongy	16	14	15	11	15	8
Odd aroma	4	6	10	24	31	41	Oily***	**3**	**3**	**4**	**4**	**19**	**21**

Terms in bold indicates differences among samples. BTF0%, BTF2.5%, BTF5%, BTF10%, BTF15%, and BTF20% means bread with tilapia flour at 0%, 2.5%, 5%, 10%, 15%, and 20% (w/w) of tilapia flour, respectively. ***P < 0.0001; **P < 0.01; *P < 0.05. ^¥ ^Evaluated in a 9-point category scale (1 = dislike extremely to 9 = like extremely). ^a─c^ Different superscripts indicate differences (*P* < 0.05) among formulations.

**Fig 2:**
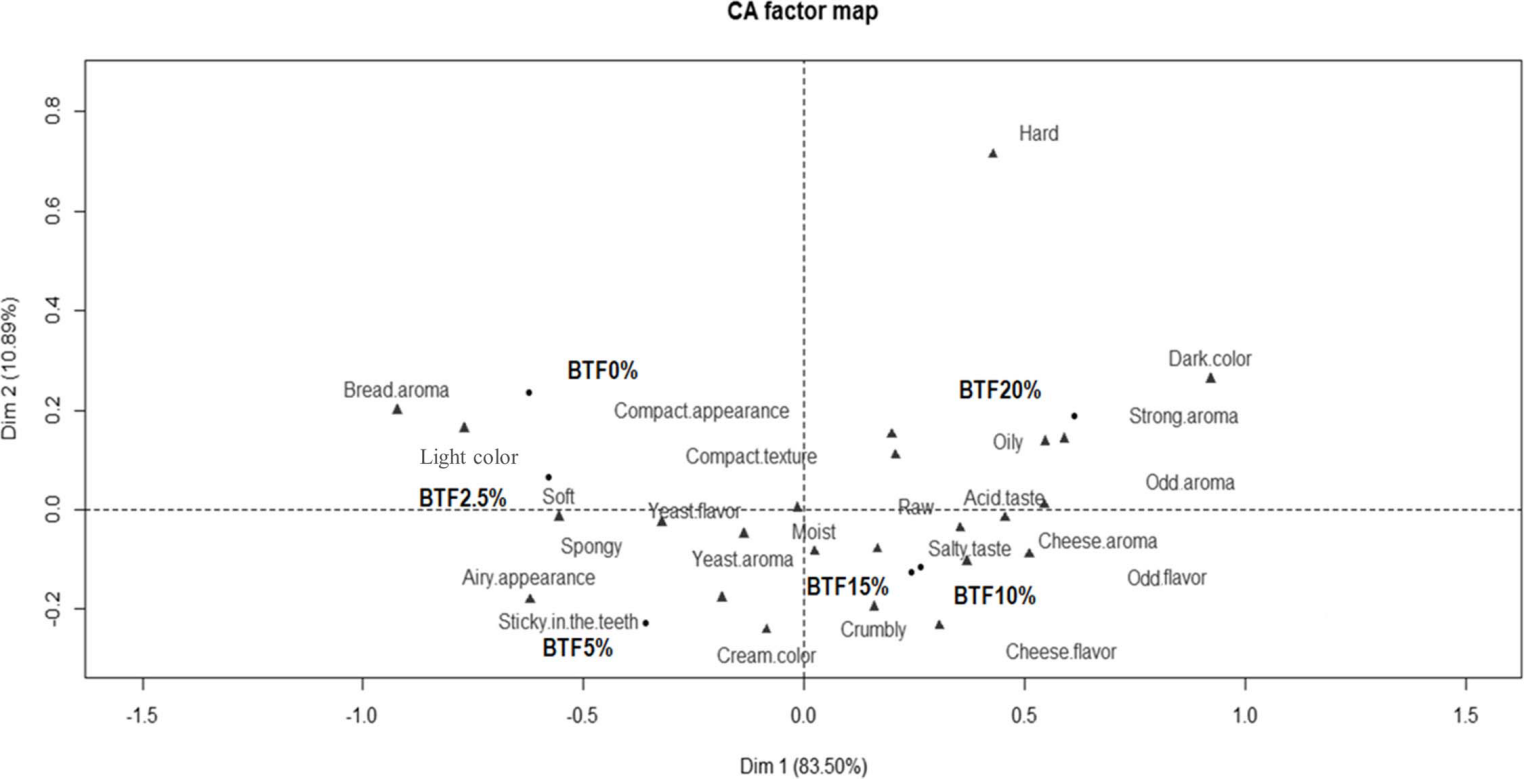
Representation of the bread samples and terms in the first and second dimension of the correspondence analysis for the BTF0%, BTF2.5%, BTF5%, BTF10%, BTF15%, and BTF20% (n = 100). BTF: bread with tilapia-waste flour at 0%, 2.5%, 5%, 10%, 15%, and 20% (w/w), respectively.

The locations of the samples and terms can be seen in the first two dimensions of the CA (Fig 2). The two dimensions explained 94.39% of the total variance (Dim 1: 83.50% and Dim 2: 10.89%) and separated the bread formulations into four groups (BTF0% and BTF2.5%; BTF5%; BTF10% and BTF15%; and BTF20%).

BTF0% and BTF2.5% were characterized mainly by the attributes *bread aroma*, *light color*, and *soft*, while BTF5% was characterized by *airy appearance*, *spongy*, *yeast aroma*, *yeast flavor*, *sticky in the teeth*, and *cream color*; however, no difference (*P* < 0.05) was observed in overall liking among BTF0%, BTF2.5%, and BTF5%. BTF10% and BTF15% were described as *moist*, *salty taste*, *acid taste*, *raw*, *cheese aroma*, *cheese flavor*, *odd flavor*, and *crumbly*. BTF20% was perceived by consumers as having *strong aroma*, *oily*, *compact texture*, *compact appearance*, *odd aroma*, *dark color*, and *hard*. To the best of our knowledge, no study has evaluated the sensory characterization of fish-enriched bread by CATA questions. Previous studies have reported sensory changes, mainly in appearance, texture, and flavor (strong taste or flavor), and decreased overall liking in bread formulations fortified with high amounts of seafood flour [8,32]. Jeyakumari et al. [9] concluded that the addition of shrimp protein hydrolysate and shrimp powder at 10% to snacks resulted in increased hardness, strong shrimp flavor, and bitterness, which in our study may have been related to the *odd* flavor. On the other hand, breads are commonly known as airy and sticky foods [33,34]. Therefore, our findings suggest that wheat flour replacement by tilapia-waste flour up to 10% was not enough to affect the sensory characteristics of the bread, and did not compromise the overall liking.

### External preference mapping

**Fig 3:**
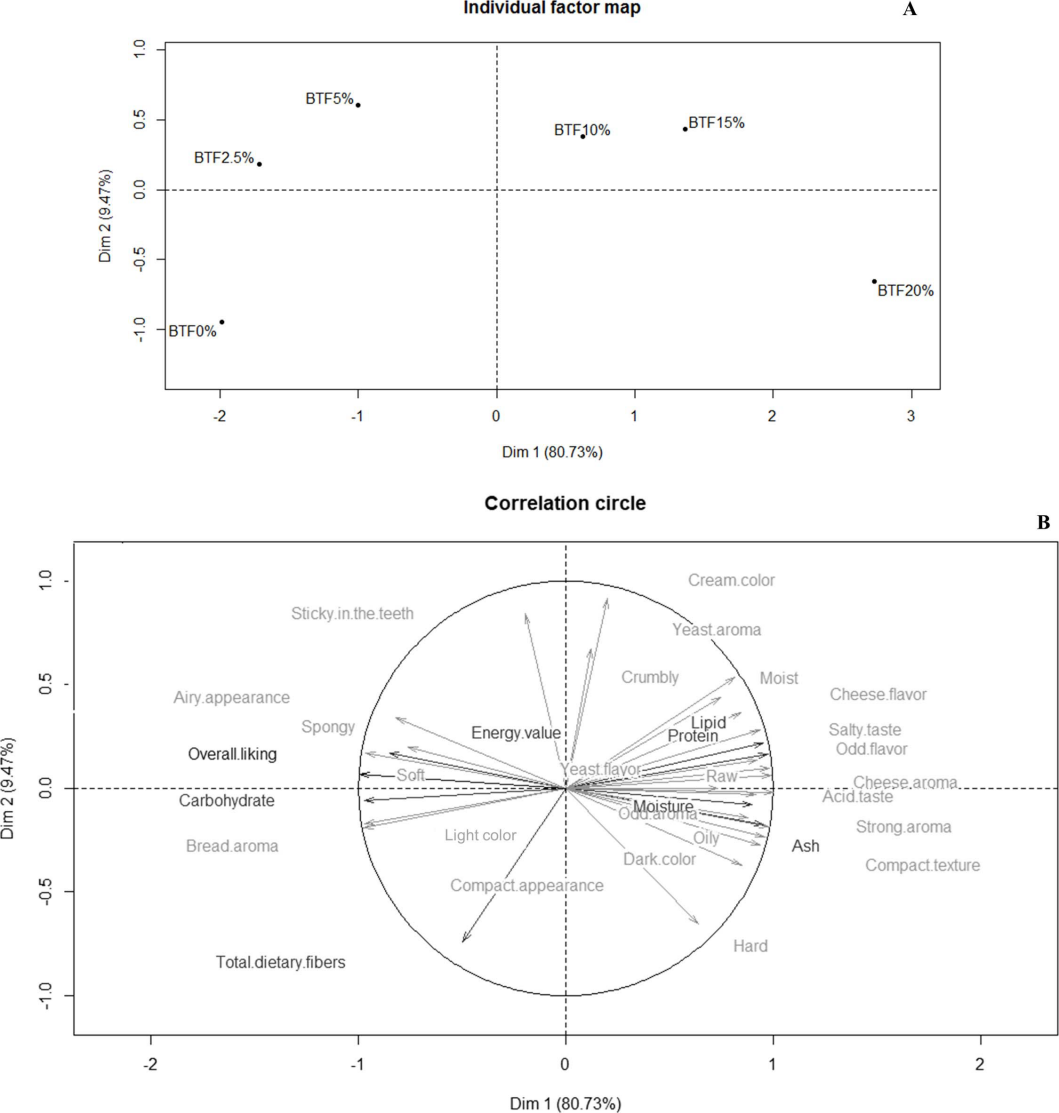
**Representation of the BTF0%, BTF2.5%, BTF5%, BTF10%, BTF15%, and BTF20% bread formulations (A) and their physical, chemical and sensory characteristics (B) provided by MFA (n = 100).** BTF: bread with tilapia-waste flour at 0%, 2.5%, 5%, 10%, 15%, and 20% (w/w), respectively.

Heenan et al. [35] described sensory characteristics to differentiate several bread types (white bread, bagel, focaccia, brioche, and ciabatta). However, consumer perceptions depended mainly on the ingredients and processing conditions such as fermentation and baking parameters [36,37], making comparison among studies difficult. In our study, the perception of oiliness in bread with high levels of tilapia flour (Fig 3) can be associated with the higher lipid content in tilapia flour compared to wheat flour, in accordance with our results for proximate composition. On the other hand, the consumers described bread containing more than 5% tilapia-waste flour as compact in appearance and texture, raw, moist, crumbly, and hard, which may be related to a starch-protein interaction and a consequently reduced dough expansion [38]. Likewise, aroma and flavor changes due to the tilapia-waste flour added to bread, such as strong aroma, odd aroma, cheese aroma, salty taste, acid taste, cheese flavor, and odd flavor can be attributed to several compounds from fish (e.g., free fatty acids and volatile sulfur compounds), which lend the fishy flavor and odor to products [29]. In addition, the color change in the enriched bread is due to color differences between the two flours, as tilapia-waste flour is visually darker than wheat flour. Previous studies observed similar sensory changes in bakery products enriched with seafood sources [9,39]. However, natural alternatives are currently available to enhance the quality of bakery products, such as texture enhancers (e.g., hydrocolloids) and flavoring agents (e.g., oleoresins and essential oils derived from spices) [40,41]. Notably, the overall liking was positively driven by *soft*, *airy appearance*, *spongy*, *bread aroma*, and *light color*, which were descriptive terms perceived for BTF0%, BTF2.5%, and BTF5% (Fig 3).

### Survival-analysis results

**Fig 4:**
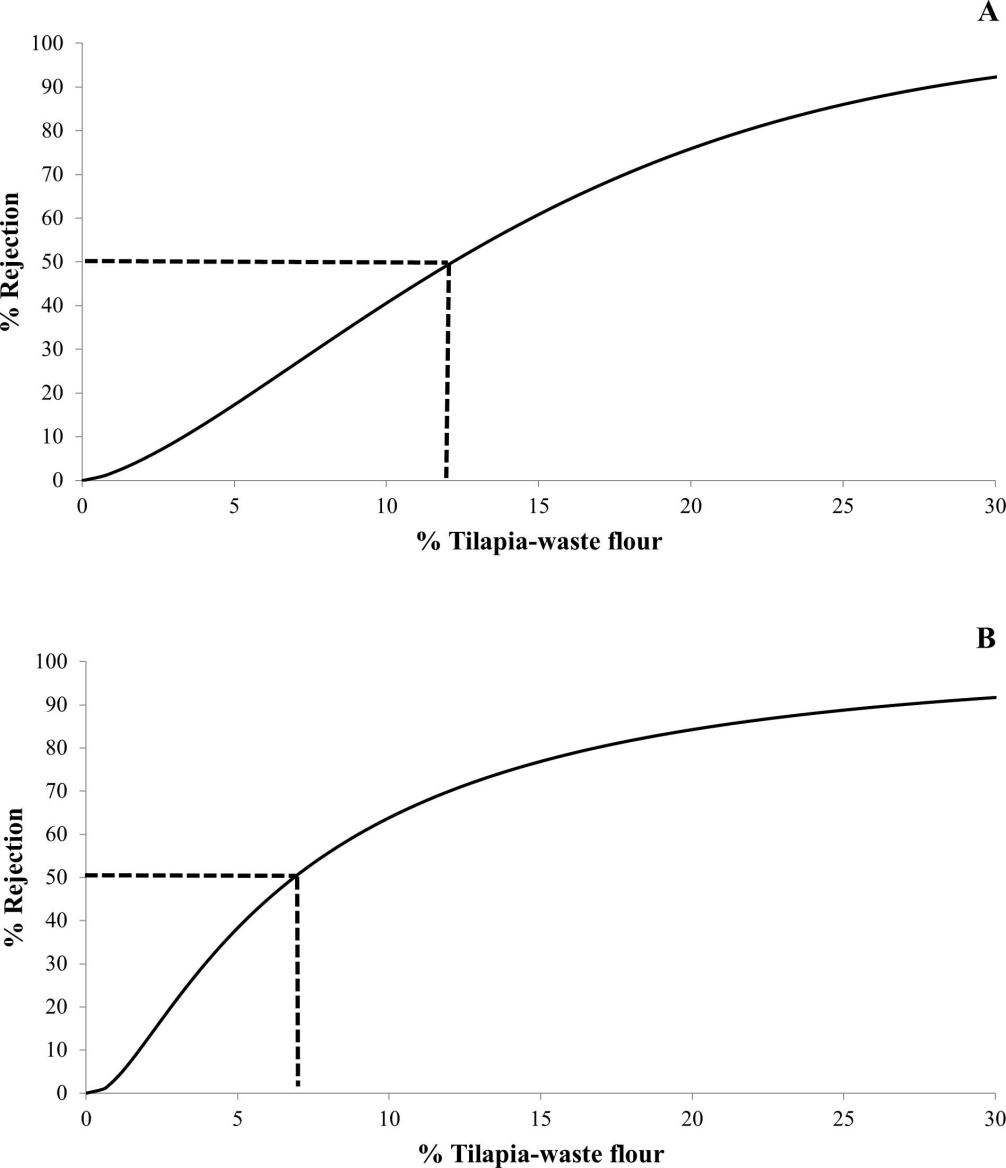
**Percentage of consumers (n = 100) that reject breads enriched with tilapia-waste flour by the Weibull (A) and lognormal (B) distributions based on answers from questions a and b.** Question a:“Suppose that you bought this product to eat or that it was served to you in your home. Would you consume it?”; Question b: “Suppose that this product is new on the market. Would you buy it?”.

Regarding the two forced-choice questions, 82%, 83%, 74%, 46%, 36%, and 21% of the participants answered “yes” to Question a, whereas 61%, 58%, 50%, 25%, 22%, and 11% replied “yes” to Question b related to BTF0%, BTF2.5%, BTF5%, BTF10%, BTF15%, and BTF20%, respectively. Fig 4 shows the percentage (%) of rejection vs. wheat flour replacement by tilapia-flour in bread with a 50% rejection probability and 5% significance level. The best fit for the data from Question a was obtained by the Weibull (μ = 2.752 and σ = 0.689) model (Fig 4A), while the log-normal (μ = 1.926 and σ = 1.062) model was used for the results from Question b (Fig 4B). Although consumer rejection increased as the amount of added tilapia flour increased, the COP value revealed that adding up to 12.17% TF did not affect the declared consumption acceptance (Fig 4A). Likewise, the intention to purchase was maintained with up to 6.86% added TF (Fig 4B).

Based on our findings, both levels of tilapia flour (12.17% and 6.86%) were enough to contribute to nutritional enrichment of bread. Tilapia-waste flour is easy to prepare, has good chemical stability at room temperature, and has a high nutritional value containing essential amino acids and fatty acids that are beneficial to human health [12,14,42]. Also, fish waste is a low-cost raw material, and its use reduces environmental impact, representing a sustainable alternative for the commercial fishing industry [11].

### Consumer interest

In response to the final question (*Would you be interested in eating bread with a higher amount of proteins and minerals*?), the majority of the consumers (72%) stated that they were interested in eating bread with higher amounts of proteins and minerals. Today’s consumers require foods that are functional, convenient, and healthy [1], which may explain the high interest in the bread with added TF. Reinforcing our findings, consumer demand is increasing for the fish matrix, more-sustainable foods, and ready-to-eat products, due to their high nutritional value, rapid preparation, and extended stability during storage [11,43]. Therefore, the substitution of wheat flour by tilapia-waste flour (≤ 10%) resulted in a convenient, sustainable and healthy product with high nutritive value (more protein, ash, lipid, and reduced energy value) without perceptible sensory changes compared to traditional breads, thereby meeting the demand of the consumer market.

## Conclusions

The replacement of wheat flour by tilapia-waste flour improved the nutritional composition of the bread. Although replacement of wheat flour by TF at or above 20% have caused changes in sensory characteristics including appearance, aroma, flavor/taste, texture, and mouthfeel, lower replacement levels (5% and 10%) could be used without detrimental effects on overall liking. In addition, our results indicated that the intention to consume and to purchase enriched bread can be maintained with levels < 12.17% and < 6.86% tilapia-waste flour, respectively. Finally, the results suggest that bread fortified with tilapia-waste flour at 5% (wheat flour replacement by 10% of TF) can be a potential alternative for the food industry to satisfy the current nutritional and sustainability requirements of consumers.

## Supporting information

S1 Table Proximate composition (%), energy value (kcal/100 g) and total dietary fibers (%) of bread formulations with different tilapia flour levels. (XLSX)
